# Valedictory Address

**Published:** 1858-05

**Authors:** Oliver Wendell Holmes


### *Valedictory Address.*

[Delivered March 10th, 1858, to the Medical Graduates of Harvard University, and communicated to the Boston Medical and Surgical Journal.]

BY OLIVER WENDELL HOLMES, M. D.

*Gentlemen of the Graduating Class*,—It is my grateful duty to address you a few words in the name of the Medical Faculty, under the auspices of which you have just entered the Medical Profession. In their name I welcome you to the labors, the obligations, the honors and the rewards which, if you you are faithful, you may look for in your chosen calling. In their name I offer you the hand of fellowship, and call you henceforth brothers. These elder brethren of the same great family repeat to you the words of welcome. The wide community of practitioners receives you in full communion from this moment. You are enrolled hereafter on that long list of the Healers of men, which stretches back unbroken to the days of Heroes and Demigods, until its earliest traditions blend with the story of the brightest of the ancient Divinities.

Once *Medicinæ Doctor*, always *Doctor Medicinæ*. You can unfrock a clergyman and unwed a husband, but you can never put off the title you have just won. Trusting that you will always cling to it, as it will cling to you, I shall venture to offer a few hints which you may find of use in your professional career.

The first counsel I would offer is this: Form a distinct PLAN for life, including duties to fulfil, virtues to practice, powers to develope, knowledge to attain, graces to acquire. Circumstances may change your plan, experience may show that it requires modication, but start with it as complete as if the performance were sure to be the exact copy of the programme. If you reject this first piece of advice, I am afraid nothing else I can say will be of service. Some weakness of mind or of moral purpose can alone account for your trusting to impulse and circumstances. Nothing else goes on well without a plan; neither a game of chess, nor a campaign, nor a manufacturing nor a commercial enterprise, and do you think that you can play this game of life, that you can fight this desperate battle, that you can organize this mighty enterprise, without sitting down to count the cost and fix the principles of action by which you are to be governed.

It is not likely that any of you will deliberately lay down a course of action pointing to a low end, to be reached by ignoble means. But keep a few noble models before you. For faithful life-long study of science you will find no better example than John Hunter, never satisfied until he had the pericardium of nature open and her heart throbbing naked in his hand. For calm, large, illuminated, philosophical intellect, hallowed by every exalted trait of character, you will look in vain for a more perfect pattern than Haller. But ask your seniors who is their living model, and if they all give you the same name, then ask them why he is thus honored, and their answers will go far towards furnishing the outline of that course I would hope you would lay down and follow.

Let us look, in the very brief space at our disposal, at some of those larger and lesser rules which might be supposed to enter as elements into the plan of a physician's life.

Duty draws the great circle which includes all else within it. Of your responsibility to the Head Physician of this vast planetary ambulance, or travelling hospital which we call Earth, I need say little. We reach the Creator chiefly through his creatures. Whoso gave the cup of cold water to the disciple gave it to the Master; whoso received that Master received the Infinite Father who sent him. If performed in the right spirit, there is no higher worship than the unpurchased service of the medical priesthood. The sick man's faltered blessing reaches heaven through the battered roof of his hovel before the Te Deum that reverberates in vast cathedrals.

Your duty as physicians involves the practice of every virtue and the shunning of every vice. But there are certain virtues and graces of preëminent necessity to the physician, and certain vices and minor faults against which he must be particularly guarded.

And first, of *truth.* Lying is the great temptation to which physicians are exposed. Clergymen are expected to tell such portions of truth as they think will be useful. Their danger is the *suppressio veri*, rather than direct falsehood. Lawyers stand in professional and technical relations to veracity. Thus, the clerk swears a witness to tell the truth, the whole truth and nothing but the truth. The lawyer is expected to get out of the witness not exactly the truth, but a portion of the truth, and nothing but the truth—which suits him. The fact that there are two lawyers pulling at the witness in different directions, makes it little better; the horses pulled different ways in that horrid old punishment of tearing men to pieces; so much the worse for the man. But this is an understood thing, and we do not hesitate to believe a lawyer—outside of the Court-room.

The physician, however, is not provided with a special license to say the thing which is not. He is expected to know the truth, and to be ready to tell it. Yet nothing is harder than for him always to do it. Whenever he makes an unnecessary visit, he tells a lie. Whenever he writes an unnecessary prescription, he tells a lie. It is audibly whispered that some of the "general practitioners," as they are called in England, who make their profit on the medicines they dispense, are apt to be too fond of giving those which can be charged at a pleasing figure in their accounts. It would be better if the patient were allowed a certain discount from his bill for every dose he took, just as children are compensated by their parents for swallowing hideous medicinal mixtures.

All false pretences whatsoever, acted or spoken; all superficial diagnoses, where the practitioner does not know that he knows, or, still worse, knows that he does not know; all unwarranted prognoses and promises of cure; all claiming for treatment that which may have been owing to Nature only; all shallow excuses for the results of bad practice, are lies and nothing else.

There is one safe rule which I will venture to lay down for your guide in every professional act, involving the immediate relation with the object of your cure; so plain that it may be sneered at as a truism, but so difficult to follow that he who has never broken it deserves canonizing better than many saints in the calendar: *A physician's first duty is to his patient; his second only, to himself.*

All quackery reverses this principle as its fundamental axiom. Every practitioner who reverses it is a quack. A man who follows it may be ignorant, but his ignorance will sometimes be safer than a selfish man's knowledge.

You will find that this principle will not only keep you in the great highway of truth, but that if it is ever a question whether you must leave that broad path, it will serve you as a guide. A lie is a deadly poison. You have no right to give it in large or small doses, for any selfish purpose connected with your profession, any more than for other selfish objects. But as you administer arsenic or strychnia in certain cases, without blame; nay, as it may be your duty to give them to a patient; are there not also cases in which the moral poison of deceit is rightfully employed for a patient's welfare? So many noble-hearted and conscientious persons have scruples about any infraction of the absolute rule of truth, that I am willing briefly to discuss and illustrate a question which will often be presented to you hereafter.

Truth in the abstract is perhaps made too much of as compared to certain other laws established by as high authority. If the Creator made the tree-toad so like the moss-covered bark to which it clings, and the larva of a *sphinx* so like the elm-leaf on which it lives, and that other larva so exquisitely like a broken twig, not only in color, but in the angle at which it stands from the branch to which it holds, with the obvious end of deceiving their natural enemies, are not these examples which man may follow? The Tibboo, when he sees his enemy in the distance, shrinks into a motionless heap, trusting that he may be taken for a lump of black basalt, such as is frequently met with in his native desert. The Australian, following the same instinct, crouches in such form that he may be taken for one of the burnt stumps common in his forest region. Are they not right in deceiving, or lying, to save their lives? or would a Christian missionary forbid their saving them by such a trick? If an English lady were chased by a gang of murdering and worse than murdering Sepoys, would she not have a right to cheat their pursuit by covering herself with leaves, so as to be taken for a heap of them? If you were starving on a wreck, would you die of hunger rather than cheat a fish out of the water by an artificial bait? If a school-house were on fire, would you get the children quietly down stairs under any convenient pretence, or tell them the precise truth and so have a rush and a score or two of them crushed to death in five minutes?

These extreme cases test the question of the absolute inviolability of truth. It seems to me that no one virtue can be allowed to exclude all others, with which in this mortal state it may sometimes stand in opposition. Absolute justice must be tempered by mercy; absolute truth by the law of self-preservation, by the harmless deceits of courtesy, by the excursions of the imaginative faculty, by the exigencies of human frailty, which cannot always bear the truth in health, still more in disease.

Truth is the breath of life to human society. It is the food of the immortal spirit. Yet a single word of it may kill a man as suddenly as a drop of prussic acid. An old gentleman was sitting at table when the news that Napoleon had returned from Elba was told him. He started up, repeated a line from a French play, which may be thus Englished—

> The fatal secret is at length revealed, and fell senseless in apoplexy. You remember the story of the old man who expired on hearing his sons were crowned at the Olympic games. A worthy inhabitant of a village in New Hampshire fell dead on hearing that he was chosen town clerk.

I think the physician may, in extreme cases, deal with truth as he does with food, for the sake of his patient's welfare or existence. He may partly or wholly withhold it, or, under certain circumstances, medicate it with the deadly poison of honest fraud. He must often look the cheerfulness he cannot feel, and encourage the hope he cannot confidently share. He must sometimes conceal and sometimes disguise a truth which it would be perilous or fatal to speak out.

I will tell you two stories to fix these remarks in your memory. When I was a boy, a grim old Doctor in a neighboring town was struck down and crushed by a loaded sledge. He got up, staggered a few paces, fell and died. He had been in attendance upon an ancient lady, a connection of my own, who at that moment was lying in a most critical position. The news of the accident reached her, but not its fatal character. Presently the minister of the parish came in, and a brief conversation like this followed.—Is the Doctor badly hurt?—Yes. Does he suffer much? He does not; he is easy.—And so the old gentlewoman blessed God and went off to sleep, to learn the whole story at a fitter and safer moment. I know the minister was a man of truth, and I think he showed himself in this instance a man of wisdom.

Of the great caution with which truth must often be handled, I cannot give you a better illustration than the following from my own experience. A young man accompanied by his young wife, came from a distant place, and sent for me to see him at his hotel. He wanted his chest examined, he told me.—Did he wish to be informed of what I might discover?—He did.—I made the *ante-mortem* autopsy desired. Tubercles; cavities; disease in full blast; death waiting at the door. I did not say this, of course, but waited for his question.—Are there any tubercles? he asked presently.—Yes, there are.—There was silence for a brief space, and then, like Esau, he lifted up his voice and wept; he cried with a great and exceeding bitter cry, and then the twain, husband and wife, with loud ululation and passionate wringing of hands, shrieked in wild chorus like the *keeners* of an Irish funeral, and would not be soothed or comforted. The fool? He had brought a letter from his physician, warning me not to give an opinion to the patient himself, but to write it to him, the medical adviser, and this letter *the patient had kept back*, determined to have my opinion from my own lips, not doubting that it would be favorable. In six weeks he was dead, and I never questioned that his own folly and my telling him the naked truth killed him before his time.

If the physician, then, is ever authorized to tamper with truth, for the good of those whose lives are entrusted to him, you see how his moral sense may become endangered. Plain speaking, with plenty of discreet silence, is the rule; but read the story of the wife of Cæcinna Pætus, with her sick husband and dead child, in the letters of Pliny the Younger (Lib.

III. XVI.), and that of good King David's faithful wife Michal, how she cheated Saul's cut-throats (1 Samuel, XIX. 13), before you proclaim that homicide is always better than *vericide.*

If you can avoid this most easily besetting sin of falsehood, to which your profession offers such peculiar temptations, and for which it affords such facilities, I can hardly fear that the closely related virtues which cling to truth, honesty and fidelity to those who trust you, will be wanting to your character.

That you must be temperate, so that you can be masters of your faculties at all times; that you must be pure, so that you shall pass the sacred barriers of the family circle, open to you as to none other of all outside world, without polluting its sanctuary by your presence,it is, I think, needless for me to urge.

Charity is the eminent virtue of the medical profession. Show me the garret or the cellar which its messengers do not penetrate; tell me of the pestilence which its heroes have not braved in their errands of mercy; name to me the young practitioner who is not ready to be the servant of servants in the cause of humanity, or the old one whose counsel is not ready for him in his perplexities, and I will expatiate upon the claims of a virtue which I am content to leave you to learn from those who have gone before you, and whose footprints you will find the path to every haunt of stricken humanity.

But there are lesser virtues, with their corresponding failings, which will bear a few words of counsel.

First, then, of that honorable reserve with reference to the history of his patients, which should belong to every practitioner. No high-minded or even well-bred man can ever forget it; yet men who might be supposed both high-minded and well-bred have been known habitually to violate its sacred law. As a breach of trust, it demands the sternest sentence which can be pronounced on the offence of a faithless agent. As a mark of vanity and egotism, there is nothing more characteristic than to be always babbling about one's patients, and nothing brings a man an ampler return of contempt among his fellows. But as this kind of talk is often intended to prove a man's respectability by showing that he attends rich or great people, and as this implies that a medical man needs some contact of the kind to give him position, it breaks the next rule I shall give you, and must be stigmatized as *leze-majesty* toward the Divine Art of Healing.

This next rule I proclaim in no hesitating accents: *Respect your own profession!* If Sir Astley Cooper was ever called to let off the impure ichor from the bloated limbs of George the Fourth, it was the King that was honored by the visit. and not the Surgeon. If you do not feel as you cross the millionaire's threshold that your Art is nobler than his palace, the footman that lets you in is your fitting companion, and not his master. Respect your profession, and you will not chatter about your "patrons," thinking to gild yourselves by rubbing against wealth and splendor. Be a little proud—it will not hurt you; and remember that it depends on how the profession bears itself whether its members are the peers of the highest, or the barely tolerated operatives of society, like those Egyptian dissectors, hired to use their ignoble implements, and then chased from the house where they had exercised their craft, followed by curses and volleys and stones. The Farther of your Art treated with a Monarch as his equal. But the Barber-Surgeon's Hall is still standing in London. You may hold yourselves fit for the palaces of princes, or you may creep back to the Hall of the Barber-Surgeons, just as you like. Richard Wiseman, who believed that a rotten old king. with the *corona Veneris* encircling his forehead with its copper diadem, could cure scrofula by laying his finger on its subject,—Richard Wiseman, one of the lights of the profession in his time, spoke about giving his patients over to his "servants" to be dressed after an operation; We do not count the young physician or the medical student as of menial condition, though in the noble humility of science to which all things are clean, or of that "entire affection" which, as Spenser tells us, "hateth nicer hands," they stoop to offices which the white-gloved waiter would shrink from performing. It is not here, certainly, where John Brooks—not without urgent solicitation from lips which still retain their impassioned energy—was taken from his quiet country rides, to hold the helm of our Imperial State; not here, where Joseph Warren left the bedside of his patients to fall on the smoking breastwork of yonder summit, dragging with him, as he fell, the curtain that hung before the grandest drama ever acted on the stage of time—not *here* that the Healer of men is to be looked down upon from any pedestal of power or opulence!

If you respect your profession as you ought, you will respect all honorable practitioners in this honored calling. And respecting them and yourselves, you will beware of all degarding jealousies and despise every unfair art which may promise to raise you at the expense of a rival.—How hard it is, not to undervalue those who are hotly competing with us for the prizes of life! In every great crisis our instincts are apt suddenly to rise upon us, and in these exciting struggles we are liable to be seized by that passion which led the fiery race-horse, in the height of a desperate contest to catch his rival with his teeth as he passed, and hold him back from the goal by which a few strides would have borne him.—But for the condemnation of this sin I must turn you over to the tenth commandment, which, in its last general clause, unquestionably contains this special rule for physicians—*Thou shalt not covet thy neighbor's patients.*

You can hardly cultivate any sturdy root of virtue but it will bear the leaves and flowers of some natural grace or other. If you are always fair to your professional brethren, you will almost of necessity encourage those habits of courtesy in your intercourse with them which are the breathing organs and the blossoms of the virtue from which they spring.

And now let me add various suggestions relating to matters of conduct which I cannot but think may influence your course, and contribute to your success and happiness. I will state them more or less concisely as they seem to require, but I shall utter them magisterially, for the place in which I stand allows me to speak with a certain authority.

Avoid all *habits* that tend to make you unwilling to go whenever you are wanted at any time. No over-feeding or drinking or narcotic must fasten a ball and chain to your ankle. *Semper paratus* is the only motto for a physician l

The necessity for *punctuality* is generally well understood by the profession in cities. In the country it is not unusual to observe a kind of testudinous torpor of motion, common to both man and beast, and which can hardly fail to reach the practitioner. Punctuality is so important, in consultations especially, to the patient as well as the practitioner, that nothing can excuse the want of it—not even having nothing to do—for the busiest people, as everybody knows, are the most punctual. There is another precept which I borrow from my wise friend and venerated instructor, the Emeritus Professor of Theory and Practice; and you may be very sure that he never laid down a rule he did not keep himself.—Endeavor always to make your visit to a patient at the same regular time, when he expects you. You will save him a great deal of fretting, and occasionally prevent his sending for your rival when he has got tired of waiting for you.

Your conduct in the sick room, in conversation with the patient or his friends, is a matter of very great importance to their welfare and your own reputation. You remember the ancient surgical precept—*Tuto, cito, jucunde.* I will venture to write a parallel precept under it, for the manner in which a medical practitioner shall operate with his tongue; a much more dangerous instrument than the scalpel or the bistoury. *Bre-*

*viter, suaviter, caute.* Say not too much, speak it gently, and guard it cautiously. Always remember that words used before patients or their friends are like coppers given to children; you think little of them, but the children count them over and over, make all conceivable imaginary uses of them, and very likely change them into something or other which makes them sick, and causes you to be sent for to clean out the stomach you have so unwittingly filled with trash; a task not so easy as it was to give them the means of filling it.

The forming of a diagnosis, the utterance of a prognosis, and the laying down of a plan of treatment, all demand certain particular cautions. You must learn them by mistakes, it may be feared, but there are a few hints which you may not be the worse for hearing.

Sooner or later, every body is tripped up in forming a diagnosis. I saw Velpeau tie one of the carotid arteries for a supposed aneurism, which was only a little harmless tumor, and killed his patient. Mr. Dease, of Dublin, was more fortunate in a case which he boldly declared an abscess, while others thought it an aneurism. He thrust a lancet into it and proved himself in the right. Soon after, he made a similar diagnosis. He thrust in his lancet as before, and out gushed the patient's blood and his life with it. The next morning Mr. Dease was found dead and floating in his own blood. He had divided the femoral artery. The same caution that the surgeon must exercise in his examination of external diseases, the physician must carry into all his physical explorations. If the one can be cheated by an external swelling, the other may be deceived by an internal disease. Be very careful; be very slow; be very modest in the presence of Nature. One special caution let me add. If you are ever so accurate in your physical explorations, do not rely too much upon your results. Given fifty men with a certain fixed amount of organic disease, twenty may die, twenty may linger indefinitely, and ten may never know they have anything the matter with them. I think you will pardon my saying that I have known something of the arts of direct exploration, though I wrote a youthful Essay on them, which, of course, is liable to be considered a presumption to the contrary. I wonld not, therefore, undervalue them, but I will say that a diagnosis which maps out the physical condition ever so accurately, is, in a large proportion of cases, of less consequence than the opinion of a sensible man of experience, founded on the history of the disease, though he has never seen the patient.

And this leads me to speak of prognosis and its fallacies. I have doomed them, and seen others doom, over and over again, on the strength of physical signs, and they have lived in the most contumacious and scientifically unjustifiable manner as long as they liked, and some of them are living still. I see two men in the street, very often, who were both as good as dead in the opinion of all who saw them in their extremity. People will insist on living, sometimes, though manifestly *moribund.* In Dr. Elder's life of Kane you will find a case of this sort, told by Dr. Kane himself. The captain of a ship was dying of scurvy, but the crew mutined, and he gave up dying for the present to take care of them. An old lady in this city, *near her end,* got a little vexed about a proposed change in her will; made up her mind not to die just then; ordered a coach; was driven twenty miles to the house of a relative, and lived four years longer. Cotton Mather tells some good stories which he picked up in his experience, or out of his books, showing *unstable equilibrium* of prognosis. Simon Stone was shot in nine places, and as he lay for dead the Indians made two hacks with a hatchet to cut his head off. He got well, however, and was a lusty fellow in Cotton Mather's time. Jabez Musgrove was shot with a bullet that went in at his ear and came out at his eye on the other side. A couple of bullets went through his body also. Jabez got well, however, and lived many years. *Per contra*, Colonel Rossiter, cracking a plum-stone with his teeth, broke a tooth and lost his life. We have seen physicians dying, like Spigelius, from a scratch; and a man who had had a crowbar shot through his head and well. These extreme cases are warnings. But you can never be too cautious in your prognosis, in the view of the great uncertainty of the course of any disease not long watched, and the many unexpected turns it may take.

I think I am not the first to utter the following caution:—Beware how you take away *hope* from any human being. Nothing is clearer than that the merciful Creator intends to blind most people as they pass down into the dark valley. Without very good reasons, temporal or spiritual, we should not interfere with his kind arrangements. It is the height of cruelty and the extreme of impertinence to tell your patient he must die, except you are sure he wishes to know it, or there is some particular cause for his knowing it. I should be especially unwilling to tell a child that it cannot recover; if the theologians think it necessary, let them take the responsibility. God leads it by the hand to the edge of the precipice in happy unconsciousness, and I would not open its eyes to what he wisely conceals.

Having settled the cautious course to be pursued in deciding what a disease is, and what its course is to be; having considered how much of your knowledge or belief is to be told, and to whom it is to be imparted, the whole question of treatment remains to be reduced to system.

It is not a pleasant thing to find that one has killed a patient by a slip of the pen. I am afraid our barbarous method of writing prescriptions in what is sometimes fancifully called Latin, and with the old astrological sign of Jupiter at the head of them to bring good luck, may have helped to swell the list of casualties. We understand why plants and minerals should have technical names, but I am much disposed to think that good plain English, written out at full length, is good enough for anybody's use. Why should I employ the language of Celsus? He commonly used none but his own. However, if we must use a dead language, and symbols that are not only dead, but damned, by all sound theology, let us be very careful in forming those medical quavers and semiquavers that stand for ounces and drachms, and all our other enlightened hieroglyphics. One other rule I may venture to give, forced upon me by my own experience. After writing a recipe, make it an invariable rule to read it over, not mechanically, but with all your faculties wide awake. One sometimes *writes* a prescription as if his hand were guided by a medium—automatically, as the hind legs of a water-beetle strike out in the water after they are separated from the rest of him. If all of you will follow the rule I have given, sooner or later some one among you will very probably find himself the author of a homicidal document, which but for this precaution might have carried out its intentions.

With regard to the exhibition of drugs as a part of your medical treatment, the golden rule is, *be sparing*. Many remedies you give would make a well person so ill that he would send for you at once if he had taken one of your doses accidentally. It is not quite fair to give such things to a sick man, unless it is clear that they will do more good than the very considerable harm you know they will cause. Be very gracious with children especially. I have seen old men shiver at the recollection of the rhubarb and jalap of infancy. You may depend upon it that half the success of Homœopathy is due to the sweet peace it has brought into the nursery. Between the gurgling down of loathsome mixtures and the saccharine deliquescence of a minute globule, what tender mother could for a moment hesitate?

Let me add one other hint which I believe will approve itself on trial. After proper experience of the most approved forms of remedies, or of such as you yourselves select and combine, make out your own brief list of real every-day prescriptions, and do not fall into the habit of those extemporaneous, fancy-combinations, which amuse the physician more than they profit the patient. Once more: if you must give a medicine, do it in a manly way, and not in half doses, hacking but not chopping at the stem of the deadly fruited tree you would bring down. Remember this, too; that although remedies may often be combined advantageously, the difficulty of estimating the effects of a prescription is as the square of the number of its ingredients. The deeper you wade in polypharmacy, the less you see of the ground on which you stand.

It is time to bring these hurried and crowded remarks to a close. Reject what in them is false, examine what is doubtful, remember what is true; and so, God bless you, Gentlemen, and Farewell!

